# Streptococcus Agalactiae Infective Endocarditis in a Healthy Middle-aged Man: Uncommon but Life-threatening

**DOI:** 10.7759/cureus.2632

**Published:** 2018-05-16

**Authors:** Lina Ya'qoub, Lyna Rehan, Shailja Parikh, Jonathan Enriquez

**Affiliations:** 1 Internal Medicine, University of Missouri - Kansas City; 2 Medical Student, University of Missouri-Kansas City; 3 Cardiology, University of Missouri - Kansas City

**Keywords:** infective endocarditis, streptococcus agalactiae

## Abstract

Streptococcus agalactiae (S.agalactiae) is known to cause invasive infections in pregnant women, newborns, and immunosuppressed patients. It is an uncommon but life-threatening case of infective endocarditis in middle-aged otherwise healthy adults. We present a case of a patient with life-threatening infective endocaritis caused by Streptococcus agalactiae, who passed away despite medical treatment.

## Introduction

Streptococcus agalactiae (S. agalactiae) or Group B Streptococcus (GBS) is a gram-positive coccus that is known to cause serious diseases. GBS most commonly causes infections in neonates, pregnant patients as well as elderly patients with significant underlying diseases such as diabetes mellitus, neurological impairment, cancer, and cirrhosis of the liver. The common presentations of S. agalactiae are skin and soft tissue infections. Meningitis and endocarditis are less common presentations, but they are associated with serious morbidity and mortality [1,2].

## Case presentation

**Figure 1:**
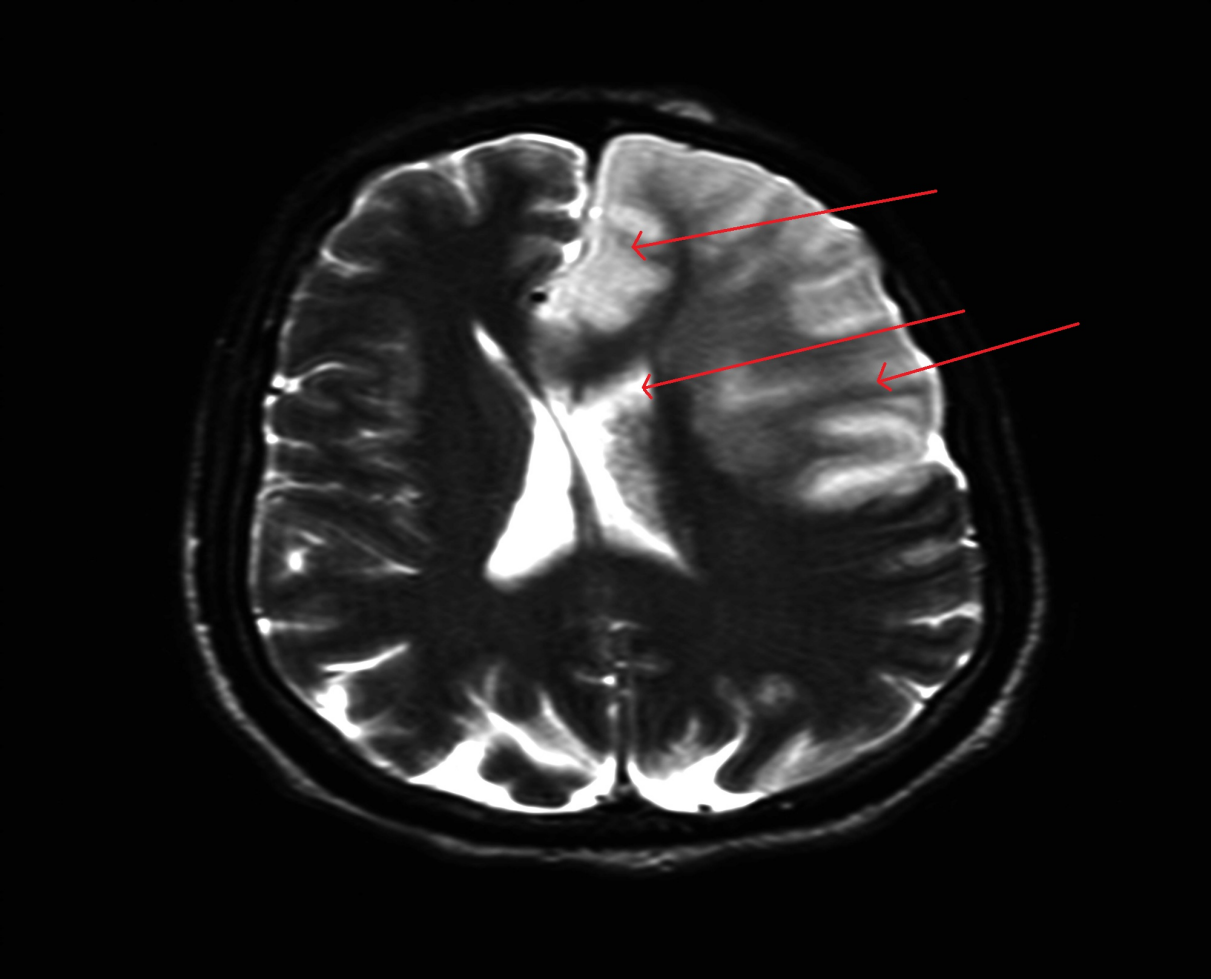
MRI of the brain showing infarction in the left frontal, left parietal, and left caudate area, concerning for cardiac embolization. There is also a midline shift of 5 mm. MRI - Magnetic Resonance Imaging.

**Figure 2:**
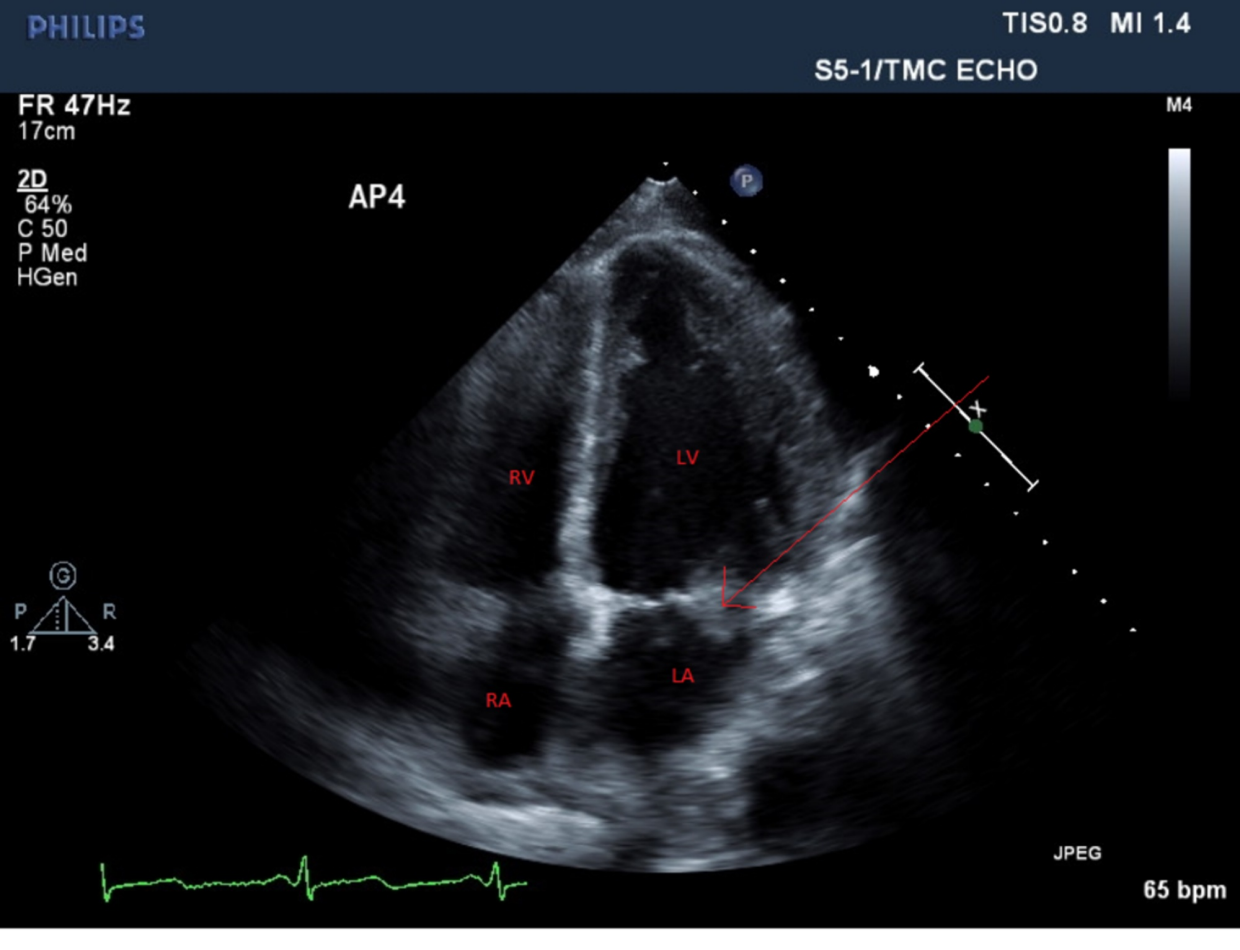
A four-chamber echocardiogram image showing a large mobile vegetation (arrow) on the mitral valve. RA: Right Atrium. RV: Right Ventricle. LA: Left Atrium. LV: Left Ventricle.

**Figure 3:**
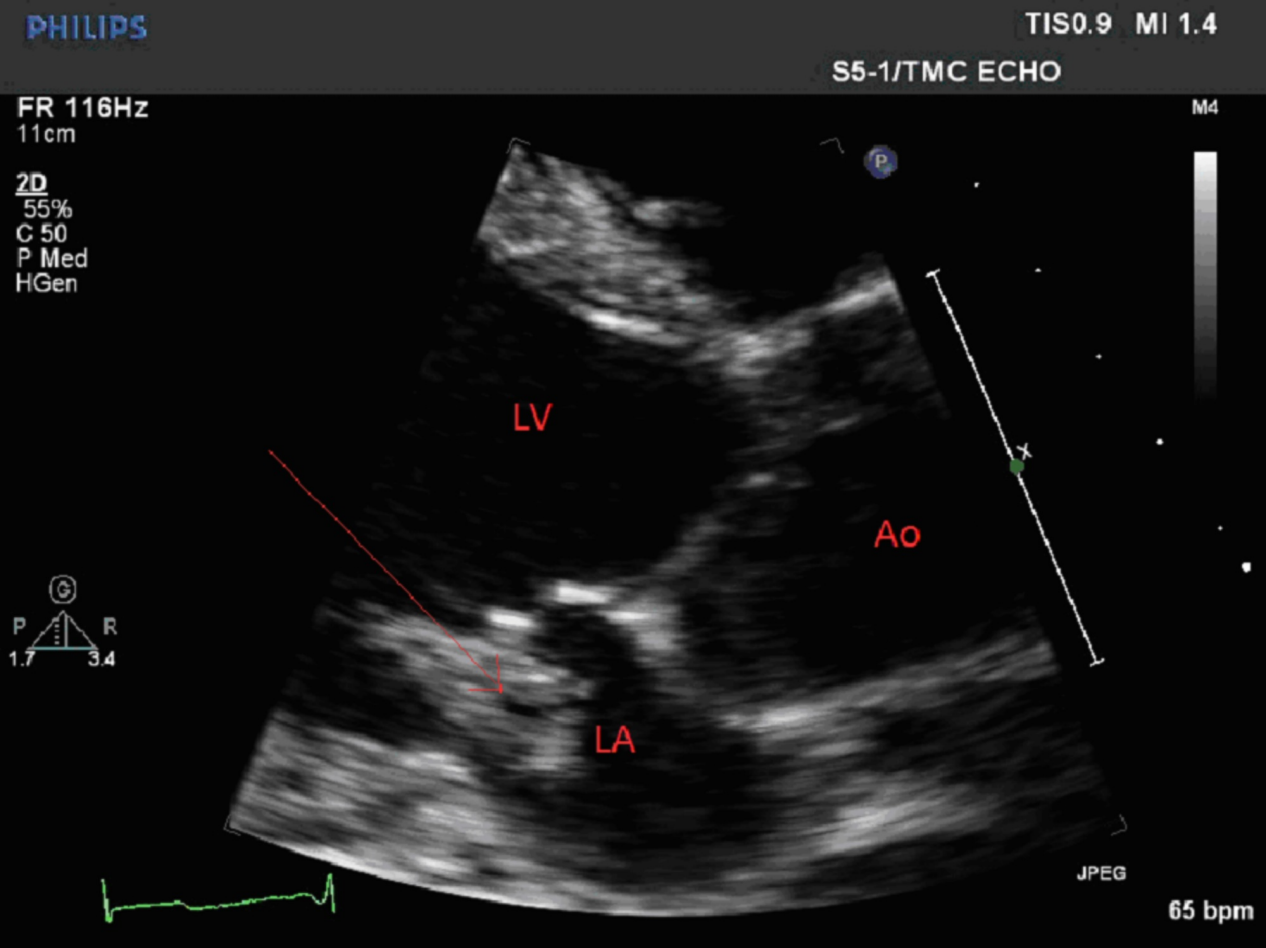
Parasternal long axis view showing the large vegetation (arrow) on the mitral valve. LA: Left Atrium. LV: Left Ventricle. Ao: Aorta.

A 48-year-old, previously healthy male was admitted to the hospital with altered mental status of one day duration. The patient was confused and was not answering questions appropriately. Vital signs were remarkable for low-grade fever of 100.7 ºF and tachycardia. His physical exam was remarkable for a holosystolic murmur at the apex, radiating to the axilla. He was alert and oriented to self, but not to place or time. Cranial nerves were grossly intact with no focal neurological deficits. Laboratory evaluation revealed leukocytosis and mild hyponatremia. A computed tomography scan of the head did not show any acute intracranial hemorrhage. A lumbar puncture was performed and cerebrospinal fluid analysis did not suggest meningitis; however, the patient was started empirically on vancomycin, ceftriaxone, ampicillin, acyclovir, and dexamethasone. Magnetic resonance imaging of the brain showed large area of infarction in the left frontal, left parietal, and left caudate body, suggestive of a cardio-embolic source (Figure 1). An echocardiogram revealed a large, mobile, vegetation (1.5 x 1.5 cm) on the mitral valve likely affecting the anterior and posterior leaflets with mild to moderate mitral regurgitation (Figures 2-3). Blood cultures were sent to the lab, which later grew Streptococcus agalactiae. An infectious disease team was consulted and antibiotics were switched to penicillin G and gentamicin.

A cardiothoracic surgery team was consulted and he was not deemed a surgical candidate as it was thought it would be unlikely that the patient will have a meaningful recovery, and the risks outweighed the benefits of surgery. The patient’s mental status remained the same; he remained alert and oriented to self only despite several days of antibiotics. His repeat blood cultures remained negative. In the third week of his hospitalization, the patient experienced worsening of his altered mental status and he was not responding to questions. An MRI of the brain showed hemorrhagic transformation of embolic infarcts with moderate cerebral edema and midline shift. He was transferred to the intensive care unit (ICU) for close monitoring. On the following day, he became unresponsive and was found to be in pulseless electrical activity. Emergency resuscitation efforts were unsuccessful. The family elected to withdraw care and the patient expired.

## Discussion

Streptococcus agalactiae is a gram-positive coccus that is commonly known to cause invasive infections in pregnant women and newborns. Infections range from local skin and soft tissue infections to invasive infections with meningitis, infective endocarditis, and sepsis [1,2]. S. agalactiae can cause invasive infections in adults with immunosuppression and chronic medical illnesses such as cancer, HIV, and diabetes [1]. However, it is an uncommon increasingly-recognized cause of infective endocarditis in patients who have no predisposing conditions [1,2]. The most common clinical presentations of S. agalactiae infection in non-pregnant patients include bacteremia without a focus and soft tissue infection. Less commonly, it causes endocarditis, urinary tract infection, and abdominal infections [3-6]. The risk factors for developing invasive S. agalactiae infections include chronic kidney disease, cardiac failure, history of neoplasia, history of ischemic heart disease, and diabetes mellitus [5]. S. agalactiae endocarditis is an aggressive disease with a significant rate of local and systemic complications [6]. Complications associated with infection by S. agalactiae include embolization, heart failure, and significant mortality [6]. Management of infective endocarditis caused by S. agalactiae is challenging. Although the microorganism is usually susceptible to beta lactam antibiotics, the minimal inhibitory concentration (MIC) tends to be higher compared to other Streptococcus species [7]. These patients are usually treated with beta-lactam antibiotics combined with gentamicin for a total of four to six weeks. Early surgery should be considered, especially in those with large vegetations > 1 cm, embolization, heart failure, and failure of medical treatment [8]. Moreover, given the high prevalence of complications and mortality associated with this microorganism, with a mortality rate as high as 56%, a multidisciplinary team consisting of a cardiologist, a cardiac surgeon, and infectious disease specialist may be advisable in the management of these patients [8].

## Conclusions

In conclusion, Streptococcus agalactiae is an uncommon but life-threatening cause of infective endocarditis in middle-aged adults without predisposing conditions. This case illustrates how aggressively S. agalactiae endocarditis can present; physicians should be aware of the exceedingly high risk of complications and mortality rate associated with this rare invasive infectious process.
